# Complement-related genetic analysis for Japanese children with transplant-associated thrombotic microangiopathy

**DOI:** 10.3389/fped.2026.1879540

**Published:** 2026-07-21

**Authors:** Ai Yamada, Shun Nagasawa, Midori Nakagawa, Sachiyo Kamimura, Naoki Sakata, Hideki Nakayama, Daiichiro Hasegawa, Yasuhiro Okamoto, Masanobu Takeuchi, Osamu Ohara, Hiroshi Moritake

**Affiliations:** 1 Division of Pediatrics, Faculty of Medicine, University of Miyazaki, Miyazaki, Japan; 2 Department of Pediatrics, Kindai University Hospital, Osaka, Japan; 3 Department of Pediatrics, National Hospital Organization Kyushu Cancer Center, Fukuoka, Japan; 4 Department of Hematology/Oncology, Hyogo Prefectural Kobe Children’s Hospital, Hyogo, Japan; 5 Department of Pediatrics, Kagoshima University School of Medical and Dental Science, Kagoshima, Japan; 6 Department of Pediatrics, Yokohama City University Hospital, Yokohama, Japan; 7 Department of Applied Genomics, Kazusa DNA Research Institute, Chiba, Japan

**Keywords:** rare C1*RL* variant, complement dysregulation, genetic susceptibility, hematopoietic stem cell transplantation, transplant-associated thrombotic microangiopathy

## Abstract

**Background:**

Transplant-associated thrombotic microangiopathy (TA-TMA) is a life-threatening complication of hematopoietic stem cell transplantation (HSCT). Previous reports in the United States have suggested that TA-TMA is caused by complement-related genetic variants. However, these findings need to be validated in other countries and ethnic populations.

**Methods:**

We performed targeted sequencing of 40 complement-and coagulopathy-related genes in 44 Japanese pediatric patients who underwent HSCT, including 20 patients with TA-TMA and 24 patients without TA-TMA. Seventeen genes reported to be related to TA-TMA were included. Additionally, 23 genes that are thought to be associated with complement system activation and coagulopathy were investigated. *CFHR*1*/CFHR*3 deletions were also examined using multiplex ligation-dependent probe amplification.

**Results:**

There was no significant difference in the percentage of patients bearing genetic variants between patients with and without TA-TMA. Furthermore, no marked differences in the percentage of patients or the average number of rare variants per patient were found between the two groups. Although we identified several rare non-synonymous variants in TA-TMA patients, we did not find any known pathogenic variants causing TA-TMA. Interestingly, a novel rare genetic variant in the *C*1*r-like protein (C*1*RL)* gene was identified in a patient with neuroblastoma who had undergone autologous HSCT, potentially associated with TA-TMA.

**Conclusion:**

In this limited number of Japanese pediatric cohort with TA-TMA, we could not find an enrichment of rare variants among 40 complement- and coagulopathy-related genes. A novel, rare variant of *C*1*RL* was identified in a single patient with TA-TMA. Further studies with larger cohorts are necessary to clarify the genetic association in Japanese patients with TA-TMA.

## 1 Introduction

Transplant-associated thrombotic microangiopathy (TA-TMA) is a serious complication of hematopoietic stem cell transplantation (HSCT) (1–5). TMA occurs after endothelial injury caused by chemotherapy, radiotherapy, severe infections, graft-vs.-host disease (GVHD), and calcineurin inhibitors. It causes microangiopathic hemolytic anemia and platelet consumption, resulting in thrombosis and fibrin deposition in microcirculation, which affects multiple organs. TMA was identified in 30%–35% of HSCT recipients and progressed to life-threatening disease in approximately half of the patients (4, 6). The incidence of TMA is likely underestimated at many transplantation centers, as some cases are mild and self-limiting without any active therapy, and severe TMA cases can be overlooked if appropriate laboratory tests are not performed, with deaths recorded as multisystem organ failure of unclear etiology (7).

The pathophysiology of TA-TMA results from a cycle of endothelial cell activation to produce a pro-coagulant state, along with the activation of antigen-presenting cells and lymphocytes, as well as activation of the complement cascade and microthrombi formation. This has led to the formulation of a “two-hit hypothesis” in which patients with either an underlying predisposition to complement activation or pre-existing endothelial injury undergo a toxic conditioning regimen causing endothelial injury, and then additional insults are triggered by medications, alloreactivity, infections, and/or antibodies (8). Thus, increased activation of complement may cause TA-TMA; however, the detailed mechanisms underlying complement activation in TA-TMA are still unclear. Jodele et al. reported that terminal complement activation at the diagnosis of TA-TMA predicts a poor survival, suggesting that complement dysregulation is a key pathway in TA-TMA pathogenesis (9, 10). Therefore, therapeutic complement inhibition by eculizumab is considered to be effective in selected cases of TA-TMA, particularly those with evidence of complement activation (11). Importantly, recent prospective multicenter clinical trials in pediatric populations have provided robust evidence supporting the efficacy of eculizumab in high-risk TA-TMA (12).

Furthermore, 17 candidate genes known to play a role in complement activation, the likely effector mechanism for vascular injury in TA-TMA, were analyzed in 34 children with TA-TMA in comparison with 43 without TA-TMA (13). Patients who had ≥3 variants among the 17 genes were found to be at risk for developing TA-TMA (13). However, the frequencies of these variants differ substantially among ethnic backgrounds, with higher frequencies reported in African American populations than in European American populations (11). Therefore, further studies in diverse ethnic populations are needed to validate these findings in children undergoing HSCT.

In the present study, we analyzed single-nucleotide variants of 40 complement- and coagulopathy-related genes in 44 Japanese children, including 20 patients with TA-TMA after HSCT. This is the first study to analyze the variant frequencies of pathogenic genes associated with thrombotic microangiopathy in Japanese children.

## 2 Materials and methods

### 2.1 Patients

Patients with TA-TMA from six institutions were enrolled in a retrospective multi-center study. This study was approved by the institutional review board of the University of Miyazaki Hospital. Data were extracted from the medical records of children who underwent HSCT at the Cooperative Research Facilities from February 1994 to September 2020. Patients diagnosed with TA-TMA using the Cho criteria, European Group for Blood and Marrow Transplantation, or Blood and Marrow Transplant Clinical Trial Network by the attending physician at each institution during the HSCT course were included (7). A retrospective review showed that 16 of the 20 patients with TA-TMA fulfilled the modified Jodele criteria (14). Medical records were reviewed, and clinical data were manually extracted.

### 2.2 Genetic analysis

Mononuclear cells were isolated from the recipients' blood or bone marrow prior to transplantation and then stored at −80 ℃ until analysis. Genomic DNA was purified from these samples using a QIAamp DNA Blood Midi Kit (Qiagen, Valencia, CA, USA). A panel of 40 genes (Supplementary Table 1) for next-generation sequencing was established by the Kazusa DNA Research Institute (15). Seventeen genes (*CFH, CFHR*1*, CFHR*3*, CFHR*4*, CFHR*5*, CD*55*, CD*59*, CD*46*, CFI, CFB, CFP, C*5*, ADAMTS*13*, CFD, C*3*, C*4*BPA*, and *THBD*) were proposed as candidate genes by Jodele et al. (13) The remaining 23 genes related to complement and coagulopathy were included in this analysis.

The observed variants were compared against the Single Nucleotide Polymorphism Database hosted by the National Center for Biotechnology Information (NCBI). Novel variants were further evaluated using laboratory-developed bioinformatics tools (11). Variants were filtered by focusing on coding, frame shift (indels), nonsense, and splicing-modifying changes. Rare variants were defined as those with a minor allele frequency of <1% in the Human Genetic Variation Database (HGVD) of the Japanese population and the Genome Aggregation Database (gnomAD), which is composed of exome and genome sequence data from individuals worldwide. The pathogenicity of the identified variants was analyzed using ClinVar, which aggregates information on the relationships between genomic variations and human phenotypes, and using tools that predict the pathogenicity of variants via *in silico* analyses [PolyPhen-2, SIFT, and combined annotation-dependent depletion (CADD)]. In addition, deletions at the CFHR3/CFHR1 loci were detected using the multiple ligation-dependent probe amplification method (MRC Holland, Amsterdam, Netherlands).

### 2.3 Western blotting

Mononuclear cells in the peripheral blood of patients with the C1r-like protein (C1RL) variant and healthy controls without the C1RL variant were obtained. Total proteins were extracted from mononuclear cells using RIPA buffer (Thermo Fisher Scientific, Waltham, MA, USA) supplemented with protease and phosphatase inhibitors. Western blotting was performed as previously described (16). Anti-C1RL (1:200, HPA011338; Sigma-Aldrich, St. Louis, MO, USA) was used as the primary antibody, and *β*-actin (1:6000, 010-27841; FUJIFILM Wako Pure Chemical Corporation, Osaka, Japan) was used as the internal reference.

### 2.4 Prediction of pathogenicity

We tested the performance of currently used computational pathogenicity predictions using CADD version 1.6, which integrates multiple annotations into one metric as the variant prediction algorithm (https://cadd.gs.washington.edu/info). The PHRED-scaled C-score ranking was selected; variants with CADD scores ≥ 15 were considered “likely pathogenic.”

### 2.5 Statistical analyses

The median (range) and frequency are reported to describe continuous and categorical variables, respectively. Differences by group for continuous and categorical variables were compared using the Mann–Whitney U test and Fisher exact test, respectively. An overall survival (OS) event was defined as death from any cause. Acute GVHD was diagnosed and graded according to established criteria (12, 13). All *P* values were two-sided. Statistical significance was set at *p* < 0.05.

All statistical analyses were performed using EZR (Saitama Medical Center, Jichi Medical University, Saitama, Japan) (14), which is a graphical user interface for R (The R Foundation for Statistical Computing, Vienna, Austria). All statistical tests were 2-sided, and significance was assessed at *P* < 0.05.

## 3 Results

### 3.1 Study demographics and patient characteristics

The characteristics of the study cohort are summarized in Table 1. There were 20 and 24 patients with and without TA-TMA after HSCT, respectively. The median age was younger (5.9 years old) in the TA-TMA than in the non-TA-TMA (9.1 years old) patients (*p* = 0.0542). Most of the patients in both groups had malignant disorders. The stem cell source was cord blood in 2 patients, autologous peripheral blood in 5 patients, and bone marrow in 11 patients in the TA-TMA group, with none of the non-TA-TMA group using stem cells from cord blood or autologous peripheral blood (*p* = 0.0116). The proportion receiving a reduced-intensity regimen in the TA-TMA group (11/20) was significantly higher than in the non-TA-TMA group (5/24) (*p* = 0.0285). All allogeneic HSCT recipients received calcineurin inhibitors for GVHD prophylaxis. The number of patients who developed severe acute GVHD grade III or IV was higher in the TA-TMA (8/18) than in the non-TA-TMA group (1/24) (*p* = 0.0064). Infectious episodes documented prior to the diagnosis of TMA and complications of SOS were not significantly different between the TA-TMA and non-TA-TMA groups (*p* = 0.0679 and 0.583, respectively). Six patients in the TA-TMA group (6/18, 33%) received eculizumab because the levels of soluble terminal complement complex C5b-9 (sC5b-9) were elevated. Patients with TA-TMA had a significantly worse OS than patients without TA-TMA (*P* = 0.0253).

**Table 1:** Clinical characteristics.

Characteristic	TA-TMA (*n* = 20)		Without TA-TMA (*n* = 24)		*p*-value
	*N*	%	*N*	%	
Male	11	55.0	12	50.0	0.771
Median age, *y* (range)	5.9	(0.2–16)	9.1	(1.0–19.0)	0.0542
Diagnosis					0.208
Malignancy	14	70.0	21	87.5	
Bone marrow failure	4	20.0	3	12.5	
Immunodeficiency	2	10.0	0	0.0	
Donor type					0.278
Related	7	35.0	12	20.0	
Unrelated	11	55.0	12	50.0	
Auto-peripheral blood stem cells	2	10.0	0	0.0	
Stem cell source					0.0116
Bone marrow	11	55.0	18	75.0	
Peripheral blood stem cells	2	10.0	6	25.0	
Cord blood	5	25.0	0	0.0	
Auto-peripheral blood stem cells	2	10.0	0	0.0	
HLA match (allo-HCT only)					0.742
Fully matched (MRD, MUD)	6	33.3	9	37.5	
Mismatched (MMRD, MMUD, haploidentical)	12	66.7	15	62.5	
Conditioning regimen type					0.0285
Myeloablative	9	45.0	19	79.2	
Reduced Intensity	11	55.0	5	20.8	
GVHD prophylaxis (CsA/Tac based)	18	100	24	100	1.0
Acute GVHD					
Grade ≤ II	10	55.6	23	95.9	0.261
Grade III/IV	8	44.4	1	4.1	0.0064
Infection					
Bacterial infection	8	40.0	6	25.0	0.342
Viral infection	3	15.0	2	8.3	0.646
Invasive fungal disease	3	15.0	1	4.1	0.316
Sinusoidal obstruction syndrome	2	10.0	1	4.1	0.583
Overall mortality at 1y after HSCT	10	50.0	4	16.7	0.0253

TA-TMA, transplant-associated thrombotic microangiopathy, HSCT, hematopoietic stem cell transplantation, HLA, human leukocyte antigen, MRD, matched related donor, MUD, matched unrelated donor, MMRD, mismatched related donor, MMUD, mismatched unrelated donor, GVHD, graft-vs.-host disease, CyA, cyclosporine, Tac, tacrolimus, HSCT, hematopoietic stem cell transplantation.

### 3.2 No association between complement-related genetic variants and TA-TMA

Among the 20 patients with TA-TMA, we identified 24 rare variants with an allele frequency <1% in both gnomAD and HGVD of the Japanese population, including 1 nonsense, 7 missense, 1 frameshift variant, and 15 synonymous variants. Among the 24 patients without TA-TMA, we identified 27 rare variants, including 2 nonsense, 8 missense, 1 splice region variant and intronic variant, 1 frameshift variant, and 15 synonymous variants. All of these rare variants were heterozygous. The non-synonymous variants are listed in Table 2.

**Table 2:** Frequency of gene variants with complement abnormalities (gnomAD < 0.01 and HGVD < 0.01).

Variants	TA-TMA (*n* = 20)		Without TA-TMA (*n* = 24)		*p*-value
	*N*	%	*N*	%	
Identified number	13	65.0	16	66.7	1.0
Variants ≥ 3	1	5.0	4	16.7	
Variants ≥ 2	9	45.0	6	25.0	
No identified	7	35.0	8	33.3	
Number of variants	24		27		
*CFH*	2		1		
*CFHR*1	0		1		
*CFHR*4	1		0		
*CFHR*5	4		0		
*CFI*	1		0		
*C*3	2		1		
*C*5	0		3		
*MCP(CD*46*)*	0		1		
*CFD*	1		1		
*CFB*	1		1		
*DAF(CD*55*)*	0		2		
*ADAMTS*13	1		3		
*THBD*	5		0		
*CD*59	2		0		
*CFP*	0		1		
*C*1*RL*^a^	1		0		
*VWF* a	3		12		
CFHR1/CFHR3 deletion(MLPA)	5/15	33.3	5/16	31.3	1.0

a Genes other than the 17 complement-related genes associated with TA-TMA.

TA-TMA: transplant-associated thrombotic microangiopathy, gnomAD: Genome Aggregation Database, HGVD: human genetic variation database, MLPA: multiple ligation-dependent probe amplification.

The percentage of patients with rare variants in the TA-TMA (13 patients, 65.0%) and non-TA-TMA (16 patients, 66.7%) groups was not significantly different (*P* = 1.0). Among the rare non-synonymous variants identified in patients with TA-TMA, three variants were predicted to be damaging by *in silico* analysis (PolyPhen-2, SIFT, and CADD): *CFH* p. Arg830Trp, *CFHR*5 p. Pro453Ser, and *C*1*RL* p. Arg321Leu (Supplementary Table 2). Only one variant predicted to be damaging was found in patients without TA-TMA (*VWF* in p. Arg507Gly) (Supplementary Table 3). *C*1*RL* p. Arg321Leu, identified in a patient who developed severe TA-TMA after autologous peripheral blood stem cell transplantation, is an extremely rare variant (allele frequency of 0.000094 in gnomAD).

*CFHR*1*/CFHR*3 deletions by multiplex ligation-dependent probe amplification were detected in 5 of 15 (33.3%) patients with TA-TMA and 5 of 16 (31.3%) patients without TA-TMA (Table 2).

### 3.3 Western blotting analyses of the novel identified C1RL variant

**Figure 1:**
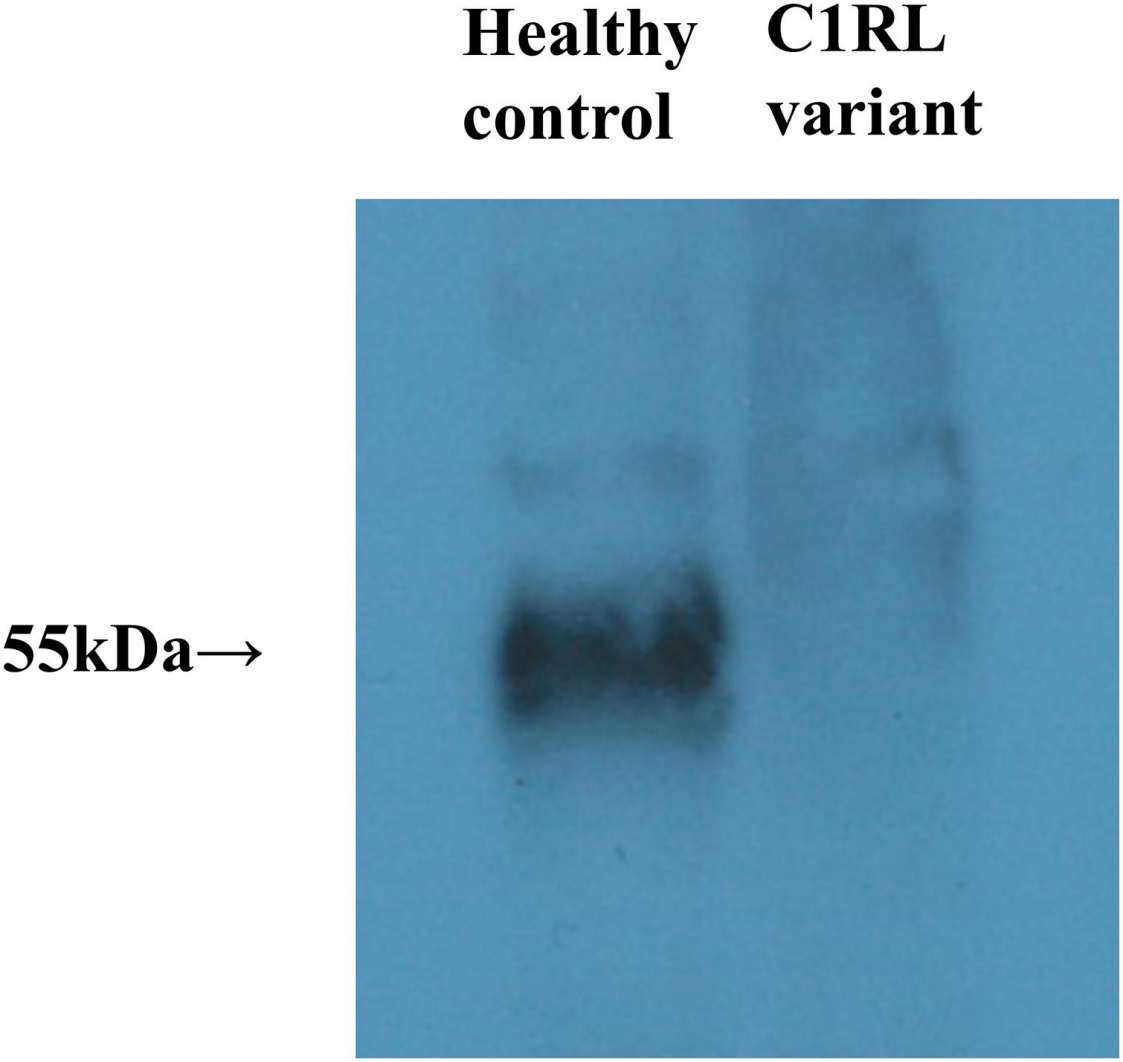
A western blot analysis of C1RL. An approximately 55 kDa C1RL protein was observed in healthy controls but was reduced in TA-TMA patients with the *C*1*RL* variant.

Western blotting demonstrated a significant reduction of the C1RL product in affected patients with the *C*1*RL* variant compared with healthy controls (Figure 1).

## 4 Discussion

In this study, we examined the frequency of rare variants of 40 complement- and coagulopathy-related genes in patients with TA-TMA and compared them with those in patients without TA-TMA. Jodele et al. reported a relationship between the number of variants in 17 complement genes and complications of TA-TMA (13); however, we did not find any association between TA-TMA development and the number of variants in these 40 genes. This discrepancy could be due to the differences in patient characteristics, as the majority reported by Jodele et al. were Americans of African and European descent, and the major underlying disease was non-malignant. All of the patients analyzed in our study were Japanese children, and many of them had malignant hematological diseases. The differences in these backgrounds might have influenced the outcome.

We detected several rare nonsynonymous variants in patients with TA-TMA. Among them, *C*1*RL* was identified as an extremely rare variant. C1RL is thought to be associated with the complement pathway through its specific function; however, its role is not fully understood. Lin et al. reported that C1RL could not cleave protease substrates but was able to inhibit complement-mediated hemolysis and other active proteases, suggesting that C1RL might play a regulatory role in the classic complement activation cascade and protease catalytic processes (17). This suggests that abnormalities or dysfunction in C1RL may affect complement regulation and lead to immune responses and inflammatory disorders. In our study, a Western blot analysis showed that the expression of C1RL protein in patients with a variant of C1RL was dramatically lower than that in healthy controls. Therefore, the reduced expression of C1RL may potentially affect complement regulation; however, this variant was identified in only one patient. To our knowledge, C1RL has not yet been reported in the context of its potential role in TA-TMA and aHUS. A large cohort is necessary to confirm whether it contributes to the complication of TA-TMA. Furthermore, more research is needed to fully understand the role and function of C1RL in the complement pathway.

The present study suggests that the genetic screening system proposed by Jodele et al., which has shown utility in other populations, may not be useful for identifying patients at high risk of TA-TMA in Japanese pediatric patients who received HSCT, especially in malignant disorders. In addition to genetic factors, our study confirmed that clinical factors such as severe GVHD were significantly associated with TA-TMA. The observed increased incidence of severe GVHD in the TA-TMA group is consistent with previous reports (10, 18), supporting the hypothesis that immune activation and endothelial damage contribute to disease development. Furthermore, six patients with TA-TMA received eculizumab owing to elevated sC5b-9 levels, indicating that complement inhibition may have therapeutic potential in select cases. An important distinction should be made between complement activation as a pathogenic amplifier of TA-TMA and inherited complement-related genetic susceptibility as a predisposing factor. In the present study, six patients with TA-TMA showed elevated sC5b-9 levels and received eculizumab, supporting the clinical relevance of complement activation in selected patients without any known pathogenic variant. These findings suggest that complement activation in TA-TMA may frequently occur as a secondary consequence of endothelial injury, severe GVHD, infection, conditioning-related toxicity, or other transplantation-associated insults rather than being primarily driven by inherited complement dysregulation. This concept is consistent with previous studies that have reported that clinically suspected complement-mediated TMA and atypical hemolytic uremic syndrome is not always associated with identifiable pathogenic variants in complement genes (4, 19, 20).

This study had several limitations. First, the statistical power to detect modest genetic effects was limited because the sample size was relatively small. Second, the cohort was clinically heterogeneous with respect to underlying diseases, stem cell sources, conditioning regimens, and transplantation types. Third, TA-TMA was diagnosed retrospectively across multiple institutions using various established criteria, including Cho, EBMT, and BMT-CTN. A retrospective review demonstrated that 16 of the 20 patients with TA-TMA fulfilled the modified Jodele criteria, supporting the overall validity of case classification. Finally, the functional significance of the identified *C*1*RL* variant remains uncertain because it was detected in only one patient.

It is important to establish specific genetic markers and detect complement-related abnormalities early to improve the diagnosis and management of TA-TMA. A deeper understanding of the genetic mechanisms underlying TA-TMA may guide the development of tailored therapeutic strategies and new treatment targets, ultimately enhancing outcomes in pediatric HSCT patients. Future studies with larger cohorts, broader gene analyses, and functional experiments are essential to clarify the role of complement regulation and its genetic underpinnings in TA-TMA.

In conclusion, in this limited Japanese pediatric cohort, we did not observe an enrichment of rare complement- and coagulopathy-related variants among patients with TA-TMA in comparison to the controls. However, the identification of a rare C1RL variant suggests a possible contribution of complement dysregulation in selected patients. Further studies are needed to better understand the genetic mechanisms underlying TA-TMA.

## Data Availability

The datasets presented in this article are not readily available because they contain identifiable genetic and clinical information and are subject to ethical restrictions. Requests to access the datasets should be directed to the corresponding author and are subject to approval by the institutional ethics committee.
