# Supplementary material for: Complement-related genetic analysis for Japanese children with transplant-associated thrombotic microangiopathy
Source: Front Pediatr. 2026 Jul 21;14:1879540. doi: 10.3389/fped.2026.1879540 (PMC13433622; doi:10.3389/fped.2026.1879540)
Supplement: Supplementary file 1 [file Table1.docx]

**Supplementary Table S1. The 40 genes examined in the panel**

| Gene Symbol | Description | Accession no. |
| --- | --- | --- |
| CD46 | CD46 molecule, complement regulatory protein | NM_172361.2 |
| THBD | thrombomodulin | NM_000361.2 |
| CFH | complement factor H | NM_000186.3 |
| CFI | complement factor I | NM_000204.3 |
| CFB | complement factor B | NM_001710.5 |
| C3 | complement component 3 | NM_000064.3 |
| C5 | complement component 5 | NM_001735.2 |
| CFD | complement factor D | NM_001928.2 |
| CFHR1 | complement factor H-related 1 | NM_002113.2 |
| CFHR3 | complement factor H-related 3 | NM_021023.5 |
| CFHR4 | complement factor H-related 4 | NM_006684.4 |
| CFHR5 | complement factor H-related 5 | NM_030787.3 |
| CFP | complement factor properdin | NM_002621.2 |
| C4BPA | complement component 4 binding protein, alpha | NM_000715.3 |
| CD59 | CD59 molecule, complement regulatory protein | NM_203330.2 |
| CD55 | CD55 molecule, decay accelerating factor for complement | NM_000574.4 |
| ADAMTS13 | ADAM metallopeptidase with thrombospondin type 1 motif, 13 | NM_139025.4 |
| SERPING1 | serpin family G member 1 | NM_000062.3 |
| CR1 | complement C3b/C4b receptor 1 | NM_000573.4 |
| PLG | Plasminogen | NM_000301.5 |
| DGKE | diacylglycerol kinase epsilon | NM_003647.3 |
| VWF | von Willebrand factor | NM_000552.4 |
| C1RL | complement C1r subcomponent like | NM_016546.3 |
| IL1A | interleukin 1 alpha | NM_000575.5 |
| IL1B | interleukin 1 beta | NM_000576.3 |
| IL12A | interleukin 12A | NM_000882.4 |
| IL12B | interleukin 12B | NM_002187.3 |
| TNF | tumor necrosis factor | NM_000594.4 |
| SERPINE1 | serpin family E member 1 | NM_000602.5 |
| SERPINB2 | serpin family B member 2 | NM_001143818.2 |
| TBXA2R | thromboxane A2 receptor | NM_001060.6 |
| ICAM1 | intercellular adhesion molecule 1 | NM_000201.3 |
| IFNG | interferon gamma | NM_000619.3 |
| PTGIR | prostaglandin I2 receptor | NM_000960.4 |
| PTGIS | prostaglandin I2 synthase | NM_000961.4 |
| VEGFA | vascular endothelial growth factor A | NM_001025366.3 |
| SELE | selectin E | NM_000450.2 |
| SELP | selectin P | NM_003005.4 |
| F3 | tissue factor (coagulation factor III) | NM_001178096.2 |
| ANGPT2 | angiopoietin 2 | NM_001118887.2 |
