# Supplementary material for: Complement-related genetic analysis for Japanese children with transplant-associated thrombotic microangiopathy
Source: Front Pediatr. 2026 Jul 21;14:1879540. doi: 10.3389/fped.2026.1879540 (PMC13433622; doi:10.3389/fped.2026.1879540)
Supplement: Supplementary file 2 [file Table2.docx]

**Supplementary Table 2. Rare variants (gnomAD<0.01 and** **HGVD<0.01 without synonymous) in patients with 　　TA-TMA**

| Gene | Reference  SNP ID | Variants | Polyphen2 | SIFT | CADD  (PHRED) | ClinVar |
| --- | --- | --- | --- | --- | --- | --- |
| CFH | rs62641696 | c.2488C>T(p.Arg830Trp) | Possibly damaging | Damaging | 19 | VUS |
| CFI | NR | c.1684_1691del(p.Val562LeufsTer3) | N/A | ND | ND | NR |
| CFB | rs117314762 | c.221G>A(p.Arg74His) | probably damaging | Not scored | 27 | Likely Benign |
| C3 | rs184455094 | c.48C>A(p.His16Gln) | Benign | Tolerated | 9 | Benign |
| CFHR4 | rs189225757 | c.991G>A(p.Glu331Lys) | Benign | Damaging | 3 | NR |
| CFHR5 | rs762904582 | c.1708T>C(p.Ter570Argext*?) | N/A | N/A | 0.06 | NR |
| CFHR5 | rs184883943 | c.1357C>T(p.Pro453Ser) | Probably damaging | Damaging | 23 | NR |
| THBD | rs41400249 | c.1208G>A(p.Arg403Lys) | Benign | Tolerated | 14 | Likely Benign |
| C1RL | rs138272943 | c.962G>T(p.Arg321Leu) | Probably damaging | Damaging | 23 | NR |

gnomAD: genome aggregation database, HGVD: human genetic variation database, TA-TMA: transplant-associated thrombotic microangiopathy, ND: no data, N/A: not available, NR: not reported, VUS: variants of uncertain significance
