# Supplementary material for: Complement-related genetic analysis for Japanese children with transplant-associated thrombotic microangiopathy
Source: Front Pediatr. 2026 Jul 21;14:1879540. doi: 10.3389/fped.2026.1879540 (PMC13433622; doi:10.3389/fped.2026.1879540)
Supplement: Supplementary file 3 [file Table3.docx]

**Supplementary Table 3. Rare variants in withtout TA-TMA patients, gnomAD<0.01, HGVD<0.01, non synosymous**

| Gene | Reference  SNP ID. | Variants | Polyphen2 | SIFT | CADD  (PHRED) | ClinVar |
| --- | --- | --- | --- | --- | --- | --- |
| C5 | NR | c.1797G>A (p.Trp599*) | N/A | ND | ND | NR |
| CFP | NR | c.1244+7G>A | N/A | ND | ND | NR |
| CD46 | rs780693519 | c.176G>A(p.Arg59Gln) | Benign | Tolerated | 0.002 | VUS |
| VWF | rs772203447 | c.2303G>A (p.Arg768Gln) | Benign | Tolerated | 23 | Likely pathogenic |
| CD55 | rs1131690771 | c.263C>A (p.Ser88*) | N/A | ND | 37 | Affect |
| CFD | NR | c.320_323dupAGCC (p.Asp109fs) | N/A | ND | ND | NR |
| ADAMTS13 | rs781927460 | c.2605G>A(p.Gly869Ser) | Benign | Tolerated | 17 | NR |
| C5 | rs56188613 | c.1352G>T (p.Gly451Val) | Benign | Tolerated | 0.1 | VUS |
| CFHR1 | rs1335153955 | c.287A>G (p.His96Arg) | Benign | Tolerated | 8 | NR |
| VWF | rs34120165 | c.7025G>A (p.Arg2342His) | Benign | Tolerated | 9 | VUS |
| CFB | rs1185480704 | c.952C>T(p.Pro318Ser) | Benign | Tolerated | 15 | NR |
| VWF | rs189409574 | c.1519A>G(p.Arg507Gly) | Probably Damaging | Damaging | 24 | NR |

gnomAD: genome aggregation database, HGVD: human genetic variation database, TA-TMA: transplant-associated thrombotic microangiopathy, ND: no data, N/A: not available, NR: not reported, VUS: variants of uncertain significance
